# Should Preventive Antifungal Treatment Be Given to Patients With Abdominal Contamination in the Intensive Care Unit?

**DOI:** 10.7759/cureus.35071

**Published:** 2023-02-16

**Authors:** Kubra Demir Onder, Aysegul Seremet Keskin

**Affiliations:** 1 Department of Infectious Diseases and Clinical Microbiology, University of Health Sciences Antalya Training and Research Hospital, Antalya, TUR

**Keywords:** empirical antifungal, prophylaxis, intra-abdominal candidiasis, candidemia, abdominal contamination

## Abstract

Background

Intra-abdominal contamination is a critical risk factor for candidemia. Because of the high mortality of candidemia and delayed results of cultures, preventive antifungal (AF) treatment can be administered. Especially in the intensive care unit (ICU), it may be necessary to determine the preventive AF approach due to the poor clinical condition of the patients. However, this practice is not standard among clinicians, and it is controversial whether it is beneficial or not. This study aimed to evaluate the effects of different AF treatment approaches (prophylactic, empirical, and culture-directed) on mortality, development of candidemia, and length of hospital stay in these patients. The primary outcome of the study was mortality, and the secondary outcomes were the development of candidemia and length of hospital stay.

Methodology

This is a retrospective, single-center, cohort study. Adult patients who were hospitalized in the ICU with the diagnosis of intra-abdominal contamination between January 1, 2017, and December 31, 2020, were reviewed retrospectively from electronic hospital records and Infectious Diseases ICU patient follow-up forms. Age, gender, comorbid diseases, the reason for hospitalization, history of surgical operation, surgical procedure type, length of hospital stay, culture results of blood and intraoperative intra-abdominal samples (pus, peritoneal fluid, abscess), type of AF agents, and mortality status of the patients were recorded. Furthermore, white blood cell (WBC) count, platelet count, C-reactive protein (CRP) level, procalcitonin (PCT) level, and serum albumin levels in blood samples taken on three different days (the day of diagnosis, the day of operation, and the day of candidemia) were examined. The patients were grouped as without AF, receiving prophylactic AF, receiving empirical AF, and receiving culture-directed AF. Additionally, the study population was evaluated by dividing it into two groups, namely, those who developed candidemia and those who did not. The patients were evaluated regarding the development of candidemia, AF treatment approach, length of hospital stay, and mortality.

Results

A total of 196 patients were included in the study. Candidemia was determined in 31.6% of the patients. Candidemia was more common in patients with a history of previous surgery and presenting with acute abdominal pain than other causes. It was determined that 70% of the patients who developed candidemia had perforation, with the most common being colonic perforation. The hospital stay was longer in patients with candidemia than without candidemia (47.9 vs. 22.4 days; p < 0.001). When empirical and prophylactic AF recipients were compared, there was no difference in mortality and length of hospital stay. Prolongation of the time to empirical treatment after perforation/leak was associated with increased candidemia (p = 0.004). Furthermore, patients with a waiting time of ≥4.5 days until surgical operation were at a higher risk of developing candidemia.

Conclusions

Although the study did not demonstrate a difference in terms of reducing mortality, it was concluded that preventive AF therapy can be administered to reduce the risk of candidemia and hospitalization duration, especially in patients with a history of previous surgical operations and abdominal contamination with a prolonged waiting period until the surgical operation.

## Introduction

Well-known risk factors for the development of invasive candidiasis are broad-spectrum antibiotics, central venous catheters, total parenteral nutrition, renal replacement therapy, neutropenia, use of implantable prosthetic devices, use of immunosuppressive agents, and gastrointestinal (GIS) surgery [1]. Especially major abdominal surgery or GIS perforation in which the GIS system is opened is a critical risk factor for the development of candidemia [2]. The mortality rate in candidiasis of intra-abdominal origin is approximately 30%, which is approximately 40% if the patient is in the intensive care unit (ICU), and this rate may increase to 60% in the presence of septic shock [3,4].

Due to the delayed diagnosis of culture-based candidemia and the high mortality of candidemia [5], prophylactic and empirical antifungal (AF) approaches are quite common in cases of abdominal contamination before candidemia occurs. It is known that in critically ill surgical patients with intra-abdominal candidiasis, the mortality risk decreases with appropriate AF treatment and adequate focus control [3].

However, there is no standard approach among clinicians regarding administering prophylactic and empirical AF in case of intra-abdominal contamination. Fluconazole and other AF prophylaxis are controversial because of the increased risk of colonization with fluconazole-reduced susceptibility strains and non-albicans strains [6-8]. Different studies of empirical AF treatment in postoperative patients in the ICU have reported different results [9,10].

There is no standard approach by infectious disease specialists in terms of AF treatment approach (whether to administer prophylaxis or empirical treatment) and drug selection for patients at high risk for intra-abdominal candidemia. In this study, patients admitted to the ICU with intra-abdominal contamination for any reason (GIS perforation, bowel obstruction, ileus or mesenteric ischemia, penetrating trauma, anastomotic leakage, tumor perforation, ulcer perforation, spontaneous perforation) were investigated retrospectively. This study aimed to evaluate the effects of different AF treatment approaches (prophylactic, empirical, and culture-directed) on mortality, development of candidemia, and length of hospital stay in these patients. The primary outcome of the study was mortality, and the secondary outcomes were the development of candidemia and length of hospital stay.

## Materials and methods

Study design and data collection

This is a retrospective, single-center, cohort study. Adult patients who were hospitalized in the ICU with the diagnosis of intra-abdominal contamination between January 1, 2017, and December 31, 2020, were reviewed retrospectively from electronic hospital records and Infectious Diseases ICU patient follow-up forms. The hospital where the study was conducted is a tertiary training and research hospital, where most surgical operations are performed in the region.

Patients whose blood cultures were taken before AF and antibiotic treatment was initiated on the day of admission were included in the study. Patients under 18 years of age and those whose blood culture was not obtained before antimicrobial therapy were excluded.

Age, gender, comorbid diseases, the reason for hospitalization, history of surgical operation, surgical procedure type, length of hospital stay, culture results of blood and intraoperative intra-abdominal samples (pus, peritoneal fluid, abscess), type of AF agents, and mortality status of the patients were recorded. Furthermore, white blood cell (WBC) count, platelet count, C-reactive protein (CRP) level, procalcitonin (PCT) level, and serum albumin levels in blood samples taken on three different days (the day of diagnosis, the day of operation, and the day of candidemia) were examined.

The patients were grouped as without AF, receiving prophylactic AF, receiving empirical AF, and receiving culture-directed AF. Additionally, the study population was evaluated by dividing it into two groups, namely, those who developed candidemia and those who did not. The hospitalization period of the patients was investigated until discharge or death.

Microbiological methods

Yeast Identification

The blood culture system BacT/ALERT (bioMérieux SA, Marcy-l'Étoile, France) was used. *Candida *species identification was based on colony morphology on CHROMagar (Becton Dickinson, Heidelberg, Germany) and the VITEK 2 system (bioMérieux SA, Marcy-l'Étoile, France).

Bacterial Identification

BacT/ALERT (bioMérieux SA, Marcy-l'Étoile, France) was used for blood cultures. Gram staining of intra-abdominal samples was performed, and 5% sheep blood agar (SBA), chocolate agar, and eosin methylene blue agar (EMB) were cultivated. The isolates were incubated at 35 ± 2°C for 18-24 hours. Identification of isolates was carried out with matrix-assisted laser desorption/ionization-time of flight mass spectrometry (bioMérieux SA, Marcy-l'Étoile, France) and Vitek 2 (bioMérieux SA, Marcy-l'Étoile, France) systems.

Definitions

Prophylactic Antifungal Treatment

This AF treatment was initiated as a result of the diagnosis of abdominal contamination, while clinical and laboratory evidence of invasive fungal infection had not yet emerged.

Empirical Antifungal Treatment

Empirical AF treatment was administered due to the development of clinical and laboratory findings (sepsis under broad-spectrum antibiotic treatment and supporting laboratory findings) in a patient with a diagnosis of abdominal contamination, while there was no culture-proven invasive fungal infection factor yet.

Culture-Directed Antifungal Therapy

This AF treatment was initiated after yeast growth was observed in blood and/or intra-abdominal samples.

Bacteremia

Bacteremia was defined as the pathogenic bacteria growth in blood culture samples taken before antimicrobial treatment in patients with abdominal contamination.

Operation Day

The day the operation was performed on patients admitted with abdominal contamination.

Time of Diagnosis

It was when the diagnosis of abdominal contamination was made clinically and radiologically.

Waiting Time

Time from symptom onset to surgical operation (days) in patients admitted with abdominal contamination.

Clinical Unresponsiveness

The persistence of clinical evidence of infection (fever, hemodynamic instability, etc.) in patients receiving AF treatment for at least three days.

Microbiological Unresponsiveness

The continuation of *Candida *growth in control blood/intra-abdominal cultures in patients receiving AF treatment for at least three days.

Mortality was evaluated as seven-day, 14-day, 30-day, and >30-day mortality within the hospitalization period overall mortality from the day of diagnosis of intra-abdominal contamination.

Statistical analysis

SPSS Statistics version 23.0 (IBM Corp., Armonk, NY, USA) was used to evaluate the study data. The conformity of the variables to the normal distribution was examined using visual (histogram and probability graphs) and analytical methods (Kolmogorov-Smirnov/Shapiro-Wilk tests). Descriptive analyses were presented using the mean and SD values for normally distributed variables and Student’s t-test was used to evaluate the difference between groups. Number and percentage values were given for the categorical variables of the patients, and the difference between the groups was evaluated by the chi-square test. The statistical significance was determined at p-values <0.05. The value of the *waiting time until the operation of the patients who applied to the clinic with abdominal contamination* in predicting the possibility of candidemia was analyzed using the receiver operating characteristics (ROC) curve. Sensitivity and specificity values of the significant cut-off value were calculated. In the evaluation of the area under the curve (AUC), it was accepted that it was statistically significant in cases where the type 1 error level was below 5%.

Ethical considerations

This study was approved by the Institutional Review Board of the Antalya Education and Research Hospital Ethics Committee (approval number: 2019-185, 15/3).

## Results

Patients and clinical characteristics

In this study, 196 adult ICU patients admitted with the diagnosis of abdominal contamination and whose blood culture was obtained before AF and antibiotics at hospitalization were included. Of the patients, 124 (63.3%) were male, and 72 were female (36.7%). The mean age of male patients was 62.2 ± 16.85 years, and it was 65.6 ± 16.62 for female patients.

The most common comorbid disease was malignancy (n = 101), and almost half were colorectal cancers (n = 48), followed by pancreatic (n = 19) and stomach (n = 18) cancers. The other most common comorbid conditions were immunosuppression (n = 62) and diabetes mellitus (n = 44). The most common cause of intra-abdominal contamination was perforation (n = 99, 50.7%). In total, 179 (91%) patients had a history of at least one surgical operation during their hospitalization. The mean total hospital stay was 30.4 ± 26.7 days (minimum: 1, maximum: 154). Candidemia was detected in 62 (31.6%) patients.

The mortality rate within seven days after diagnosis was 10% (n = 20), the 14-day mortality rate was 16.3% (n = 32), the 30-day mortality rate was 27% (n = 53), and the crude mortality rate developing at any time during hospitalization due to contaminated abdomen was 40% (n = 79). The mean day of mortality after diagnosis was 27.8 ± 28.6 (minimum: 1, maximum: 153, median: 16) days. Demographic and clinical characteristics are presented in Table 1.

**Table 1:** Demographic and clinical characteristics. GIS = gastrointestinal system; AF = antifungal

Comorbidities	n	%
Malignancy	101	51.5
Immune suppression	62	31.6
Diabetes mellitus	44	22.4
Being a chronic care patient	24	12.2
Chronic lung disease	20	10.2
Chronic kidney disease	17	8.6
Cerebro vascular event	10	5.1
Obesity	8	4
Chronic liver disease	4	2
Reason for hospitalization		
Malignancy Surgery	38	19.4
Ileus	36	18.4
GIS bleeding/Ulcer perforation	28	14.3
Acute abdomen in a patient with a history of surgery	27	13.8
Tumor perforation	20	10.2
Mesenteric ischemia	15	7.7
Necrosis/Perforated cholecystitis	8	4.1
Trauma (beating, fall from height, traffic accident)	7	3.6
Obesity surgery	5	2.6
Sharp object injuries	5	2.6
Necrosis/Perforated appendicitis	5	2.6
Diverculitis perforation	2	1.0
Total	196	100.0
Perforation/leak level		
No perforation	39	19.9
Colon-rectum-cecum-sigmoid	62	31,6
Small intestine	34	17,3
Stomach	33	16,8
Duodenum	17	8,7
Gallbladder	8	4,1
Esophagus	3	1.5
Total	196	100.0
Presence of surgical operation during hospitalization	179	91.3
Type of surgical operation		
Small intestine resection	18	10.1
Whipple operation	17	9.5
Total gastrectomy	13	7.3
Obesity surgery	4	2.2
Peritoneal debridement	36	20.1
The primary suture in peptic ulcer perforation	18	10.1
Cholecystectomy	7	3.9
Colectomy	34	19.0
Colon repair	10	5.6
Small intestine repair	19	10.6
Appendectomy	2	1.1
Esophagectomy	1	0.6
Total	179	100
Intra-abdominal infection focus		
Perforation	99	50.7
Anastomotic leak	46	23.3
Bacterial translocation	32	16.3
Abscess	19	9.7
Total	196	100
Candidemia		
Candidemia in pre-antifungal blood culture	52	26.5
Those who did not have candidemia at first but candidemia under AF	10	5.1
Total candidemia	62	31.6

Microbiological outcomes

Candidemia was detected in 62 (31.6%) of 196 patients admitted with abdominal contamination. Of these, 52 grew in blood cultures taken before AF and 10 during follow-up while under AF. Candidemia developed in four of 33 patients who were never administered AF. It was observed that the blood cultures taken previously of these patients resulted after they died. In the study, the most common candida types causing candidemia were *C. glabrata* (35.4%), *C. albicans* (27.4%), and *C. parapsilosis* (14.5%), respectively.

Blood cultures were obtained from 196 patients and intraoperative intra-abdominal sample cultures were obtained from 104 patients. In the general population, the isolation rate of microorganisms in pretreatment blood cultures was 31.6% and in intraoperative intra-abdominal sample cultures was 66.3%. Yeast could also be isolated in intra-abdominal sample cultures in six of 62 patients with candidemia. In four of these six patients, *Candida *species grown in blood and intra-abdominal samples were the same. Microorganisms grown in pretreatment blood and intra-abdominal specimens cultures are summarized in Table 2.

**Table 2:** Microorganisms grown in intra-abdominal sample cultures and blood cultures taken intraoperatively before treatment. ESBL = extended-spectrum beta-lactamases

Reproducing factor in intra-abdominal sample	Number (n)	Percentage (%)
E. coli		
ESBL (+)	12	17.3
ESBL (-)	9	13.1
*Candida *spp.	10	14.4
*Pseudomonas *spp.	8	11.4
*Klebsiella *spp.		
ESBL (+)	6	8.9
ESBL (-)	3	4.4
*Enterococcus *spp.	5	7.3
*Acinetobacter *spp.	4	5.8
Mix factor	5	7.3
Other	7	10.1
Total	69	100
Factor grew in blood culture		
*Candida *spp.	52	69.3
*Klebsiella *spp.	7	9.4
*Enterococcus *spp.	5	6.6
E. coli	4	5.3
*Acinetobacter *spp.	2	2.7
*Staphylococcus *spp.	2	2.7
Other	3	4.0
Total	75	100

Bacteremia and antibiotic therapy

Bacteremia was detected in 23 of 196 (11.7%) patients. In total, 15 patients had bacteremia accompanying candidemia. Total hospitalization duration was longer in patients with the coexistence of candidemia and bacteremia than in patients with only candidemia, although it was not statistically significant (59.8 ± 38.9 vs. 44.8 ± 29.4 days, p = 0.132). Bacteria could also be isolated from intra-abdominal samples in nine (39.1%) of 23 patients with bacteremia. The type of bacteria grown in blood and intra-abdominal samples were the same in seven of these nine patients.

All patients in the study received antibiotics after their cultures were taken after hospitalization. The antibiotics used were imipenem (n = 53), ceftriaxone + metronidazole (n = 39), piperacillin-tazobactam (n = 27), meropenem + linezolid (n = 19), meropenem (n = 13), meropenem + daptomycin (n = 11), piperacillin-tazobactam + daptomycin (n = 9), tigecycline (n = 8), meropenem + tigecycline (n = 3), ertapenem + linezolid (n = 2), and ampicillin-sulbactam.

Evaluation of patients according to the presence of candidemia

Candidemia was detected in 62 (31.6%) of 196 patients admitted with abdominal contamination. When candidemia rates were evaluated according to the reasons for hospitalization, the rate of candidemia (both in blood cultures taken before AF and total candidemia) was significantly higher in patients with a history of previous surgery and presenting with acute abdominal pain, and the difference was statistically significant (p = 0.010 and p = 0.001, respectively). The rates of candidemia according to the reasons for hospitalization are presented in Table 3.

**Table 3:** Candidemia rates according to hospitalization reasons. *: (χ^2^ = 24.886; p = 0.010); **: (χ^2^ = 30.229, p = 0.001); AF: antifungal; GIS = gastrointestinal system

Reasons for hospitalization	Before AF candidemia growth		Total candidemia growth	
	n	%	n	%
Trauma (battery, fall from height, traffic accident)	-	-	-	-
Mesenteric ischemia	4	26.7	4	26.7
Diverculitis perforation	-	-	1	50.0
GIS bleeding/Ulcer perforation	10	35.7	11	39.3
Ileus	11	30.6	12	33.3
Sharp object injuries	-	-	-	-
Malignancy surgery	11	28.9	14	36.8
Obesity surgery	-	-	1	20.0
Necrosis/Perforated cholecystitis	-	-	-	-
Tumor perforation	2	10.0	2	10.0
Necrosis/Perforated appendicitis	-	-	-	-
Acute abdomen after previous operation history	14	51.9*	17	63.0**

There was no significant relationship between the type of surgical operation and the development of candidemia. In the study, no significant relationship was determined between the presence of comorbid disease and the development of candidemia. No significant correlation was observed between the type of malignancy and the presence of candidemia.

Perforation was detected in 44 (70.9%) of 62 patients with candidemia. When evaluated according to the perforated organ, there was a perforation in colon-rectum-cecum-sigmoid in 21 (47.7%) patients, stomach in 10 (22.8%), small intestine in eight (18.2%), duodenum in four (9%), and esophagus in one (2.3%) patient. Although the rate of candidemia was highest in colon-rectum-cecum-sigmoid perforation, the difference was not statistically significant (p = 0.440).

The patients were divided into two groups, namely, those with and without candidemia, and their laboratory parameters were examined. There was no significant difference between the groups in terms of laboratory parameters. The WBC, platelet, CRP, PCT, and albumin levels of blood tests taken on the day of yeast growth in the blood, the day of AF initiation, and the day of operation are presented in Table 4.

**Table 4:** Laboratory parameters. AF = antifungal; WBC = white blood cell; PCT = procalcitonin; CRP = C-reactive protein; PLT = platelet

	Candidemia growth		P-value
	Yes	No	
Albumin (mg/dL)			
AF start day	2.3 ± 0.4	2.4 ± 0.5	0.189
Day of operation	2.4 ± 0.5	2.6 ± 0.6	0.098
Day of yeast growth	2.4 ± 0.3	-	-
WBC (×10^3^/mm^3^)			
AF start day	11,410.6 ± 8,727	11,398.9 ± 7,898.6	0.993
Day of operation	13,153.0 ± 9,248	10,851.5 ± 6,971.4	0.076
Day of yeast growth	11,974.2 ± 9,586.5	-	-
PCT (ng/mL)			
AF start day	8.5 ± 19.8	13.6 ± 23.3	
Day of operation	17.3 ± 26.1	10.3 ± 20	0.425
Day of yeast growth	6.8 ± 17.5	-	0.318
CRP (g/L)			
AF start day	166.8 ± 106.5	207.6 ± 113.4	0.026
Day of operation	157.5 ± 121.4	170.7 ± 119.3	0.514
Day of yeast growth	150.4 ± 87.2	-	-
PLT (/mm^3^)			
AF start day	208,810.3 ± 111,598.3	243,813.7 ± 144,460.6	0.113
Day of operation	268,734.7 ± 117,233.1	270,428.6 ± 144,930.7	0.942
Day of yeast growth	224,483.8 ± 146,983.3	-	-

While the total hospital stay was 22.4 ± 19.3 days in patients who did not develop candidemia, this period was significantly prolonged at 47.9 ± 31.9 days (p < 0.001) in those with candidemia. When the patients were divided into two groups, namely, those with and without candidemia, no statistically significant difference was observed between the groups regarding general mortality rates at seven, 14, and 30 days and during hospitalization.

Evaluation of patients according to the antifungal treatment approach

In total, 67 (34.2%) patients had prophylactic AF, 72 (36.7%) had empirical AF, 24 (12.3%) had culture-directed AF (blood and/or intraabdominal), and 33 (16.8%) patients did not receive any AF. Fluconazole was the most commonly used AF agent both in prophylactic and empirical AF treatment (85.1% and 73.1%, respectively, p = 0.001). It was determined that 44 (31.6%) of 139 treatments started prophylactically and empirically required AF change due to clinical or microbiological unresponsiveness. In the prophylaxis group, of the 25% who underwent AF change, 54% were in the empirical treatment group. Echinocandins were the most preferred AF when treatment change was required, and micafungin (19/44) was used the most.

The patients were divided into four groups, namely, those who received no AF, those who received prophylactic AF, those who received empirical AF, and those who received culture-directed AF. The difference between the groups in terms of candidemia rate, seven-day mortality, 14-day mortality, 30-day mortality, mortality at any time during hospitalization, and total hospital stay was examined. There was no difference between the groups in terms of mortality, except for candidemia rate and length of stay. When the prophylactic and empirical treatment groups were evaluated for the presence of candidemia (before AF), it was observed that candidemia was higher in the empirical treatment group than in the prophylaxis group, and the difference was statistically significant (43.5% vs. 11.3%, respectively, p < 0.001). The evaluation of AF treatment groups in terms of candidemia, mortality, and length of stay is summarized in Table 5.

**Table 5:** Evaluation of AF treatment groups in terms of candidemia, mortality, and length of stay. AF = antifungal

	Not receiving AF	Prophylactic AF	Empirical AF	Culture-directed AF	P-value
Candidemia, n (%)	4 (6.5)^a^	7 (11.3)^a^	27 (43.5)^b^	24 (38.7)^c^	<0.001
Mortality, n (%)	10 (12.7)	27 (34.2)	30 (38.0)	12 (15.2)	0.500
7 days, n (%)	3 (15.0)	11 (55.0)	6 (30.0)	-	0.118
14 days, n (%)	4 (12.5)	15 (46.9)	12 (37.5)	1(3.1)	0.184
30 days, n (%)	8 (15.1)	19 (35.8)	24 (45.3)	2(3.8)	0.117
Length of stay, mean ± SD	15.2 ± 10.3^a^	22.3 ± 19.8^a^	34.8 ± 22.9^b^	61.4 ± 39.6^b^	<0.001

Time to start antifungal treatment

The mean time elapsed from the surgical operation with abdominal contamination to the prophylactic AF was 1.5 ± 1.6 days. No significant correlation was determined between the time from the operation to prophylactic AF initiation with candidemia, seven-day mortality, 14-day mortality, and 30-day mortality rates (p = 0.102, p = 0.850, p = 0.557, and p = 0.353, respectively). The mean time from the onset of perforation/leak to the onset of prophylactic AF was 4.9 ± 4 days. No significant correlation was observed between this period with candidemia, seven-day mortality, 14-day mortality, and 30-day mortality rates (p = 0.550, p = 0.782, p = 0.967, and p = 0.677, respectively).

The mean time between the operation performed with abdominal contamination and giving empiric AF was 7.6 ± 9.5 days. It was determined that the time from the operation to the initiation of empirical treatment in patients with candidemia was longer than those without candidemia, and the difference was statistically significant (12.7 ±11.8 vs. 4.7 ± 6.4 days, respectively, p = 0.001). The mean time from the onset of perforation/leak to the onset of empirical AF was 9.4 ± 9.5 days. This period was longer in those with candidemia than those without, and the difference was statistically significant (13.5 ± 11.8 vs. 6.8 ± 6.7 days, respectively, p = 0.004).

Relationship between waiting time and candidemia

**Figure 1:**
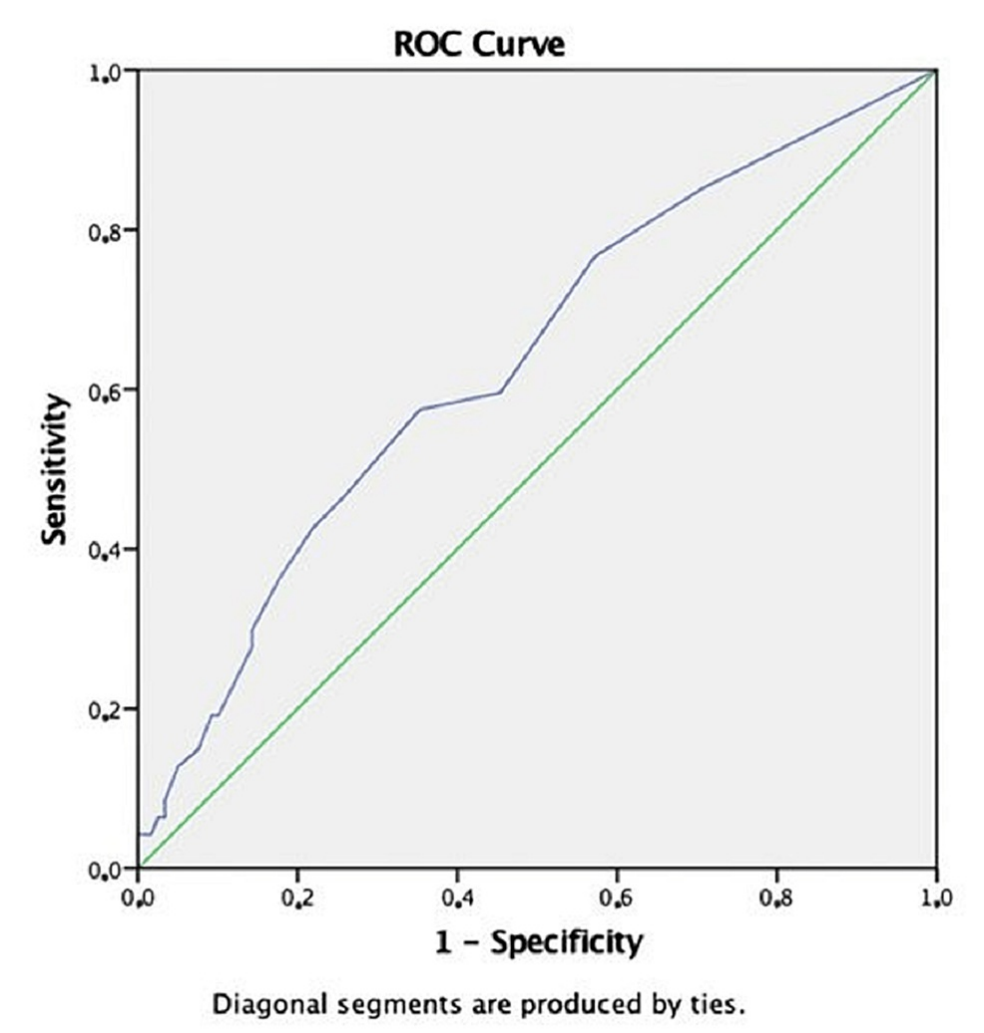
Relationship between abdominal contamination and waiting time and candidemia. ROC = receiver operating characteristics

The relationship between the time from symptom onset to the surgical operation (waiting time) and the development of candidemia was examined. The average waiting time was 7.8 ± 9.50 days in those with candidemia in pre-AF blood cultures and 7.8 ± 8.97 days in those with total candidemia. The waiting period in patients who developed candidemia (pre-AF candidemia and total candidemia) was longer than in patients who did not, and the difference was statistically significant (p = 0.013 and p = 0.005, respectively). From the ROC analysis, it was determined that patients with intra-abdominal contamination and those with a waiting time of 4.5 days or more until the operation were at a higher risk for the development of candidemia (AUC (95%): 0.636, sensitivity: 65.5%, and specificity: 38.4%). The relationship between waiting time with abdominal contamination and candidemia is shown in Figure 1.

There was no significant correlation between the waiting time until the surgical operation and the crude mortality rates at 7, 14, and 30 days and at any time during hospitalization (p = 0.422, p = 0.505, p = 0.531, and p = 0.970, respectively). When the *Candida *species and the waiting time was compared in patients with candidemia, the waiting time was 6.3 ± 3.5 days in *C. albicans*, 24 ± 26.8 days in *C. tropicalis*, 6.1 ± 6 days in *C. glabrata*, 2.3 ± 0.5 days in *C. crusei*, 8.6 ± 4.5 days in *C. parapsilosis*,and 44 days in *C.kefyr*. It was determined that the non-albicans species reproduced more in patients with a long waiting period (p < 0.001). The waiting time between abdominal contamination and the operation was longer in patients with candidemia and bacteremia than in patients with only candidemia (15.3 ± 17.76 vs. 6.2 ± 4.97, p = 0.007).

## Discussion

Preventive AF approaches in patients presenting with intra-abdominal contamination vary among clinicians. Although there have been many different clinical studies on this subject in the literature, the balance between *candidemia and mortality risk* and the *development of antifungal resistance, cost, and drug toxicity* constantly changes. In this study, the patients admitted to the ICU with the diagnosis of abdominal contamination were evaluated for candidemia, preventive AF treatments, and mortality.

The total length of hospital stay in patients with candidemia was approximately twice as long as those without candidemia. There was no statistically significant difference between patients with and without candidemia in terms of 7, 14, and 30 days and overall mortality rates. The patients were examined by dividing them into four groups, namely, those who received no AF, those who received prophylactic AF, those who received empirical AF, and those who received culture-directed AF. There was no difference between the groups in mortality, except for candidemia rate and length of stay. The prolongation of the time from the operation and the occurrence of intra-abdominal leak to the onset of empirical AF were associated with an increased risk of candidemia. It was demonstrated that candidemia increased as the waiting time until the operation increased while in the abdominal contamination clinic. However, no significant relationship was observed between waiting time and mortality.

The present study detected candidemia in 31.6% of the entire patient population. In the study by Yan et al., in a similar patient group, the rate of candidemia among all patients was 20.7% [3], and in the multicenter study by Bassetti et al., this rate was 14% [4]. When the agents of candidiasis were examined, in a study conducted between 2011 and 2013,* C. albicans* was the most common, with a rate of 64%, while *C. glabrata* could be isolated at a quarter of this rate [4]. It is known that non-albicans pathogens have increased over the years. The most common *Candida *species causing candidemia in our study was *C. glabrata* (35.4%), followed by *C. albicans* (27.4%) and *C. parapsilosis* (14.5%).

While the presence of malignancy is among the host risk factors for intra-abdominal infections [11], it is also listed among the clinical factors predicting the failure of source control in intra-abdominal infection [12]. In our study, the most common comorbid disease was malignancy (n = 101), almost half of which was colorectal cancers (n = 48). However, no significant relationship was determined between the type of malignancy and the presence of candidemia.

It is well known that upper GIS anastomotic leaks, especially gastroduodenal anastomotic leaks, including the esophagus, pose a critical risk for invasive candidiasis [1]. Treatment of AF in lower GI perforation or leakage remains controversial [13]. This study demonstrated that 70% of the patients who developed candidemia had perforation, and half of the had colonic perforation. Although there was no statistically significant difference, the rate of candidemia was observed to be the highest in colon perforation. This result suggests that in the case of perforation of the colon, which is the GIS segment with the highest concentration of anaerobic bacteria, the risk of candidemia, as well as bacteremia, should be considered.

In our study, the mortality rate within seven days after diagnosis was 10% (n = 20), the 14-day mortality rate was 16.3% (n = 32), the 30-day mortality rate was 27% (n = 53), and the crude mortality rate developing at any time during hospitalization was 40% (n = 79). The mortality rates were similar to the literature. In the study by Bassetti et al., overall 30-day hospital mortality was 27%, and this rate was reported as 38.9% in the ICU [4], while it was reported as 30.5% in the study of Yan et al. [3].

In this study, the causative microorganism isolation rate was 31.6% in blood cultures before treatment, and this rate was 66.3% in intra-abdominal sample cultures taken intraoperatively. Various studies have reported that the presence of *Candida *in intra-abdominal samples is also associated with increased mortality [14,15].

The Infectious Diseases Society of America (IDSA) guideline recommends AF treatment if *Candida *species are isolated in intra-abdominal sample cultures taken during the operation [1]. According to the Italian guideline, it is recommended to initiate AF when *Candida *growth is detected in the cultures taken from intraoperative and/or drainage catheters within the first 24 hours; but not to be treated if there is growth in the cultures taken from the drain after this period [16]. In our study, six of 62 candidemia patients (9.7%) had *Candida *growth in intraoperative intra-abdominal sample cultures. This result indicates that both intra-abdominal samples and blood cultures should be taken before the treatment in patients presenting with abdominal contamination.

Sepsis due to bacterial agents is also a vital problem in patients presenting with intra-abdominal contamination. Our study detected bacteremia in 23 (11.7%) of 196 patients. Fifteen patients had bacteremia accompanying candidemia. It was observed that the total hospitalization period was longer in patients with the coexistence of candidemia and bacteremia than in patients with only candidemia. While bacteremia was detected in only 11% of patients in the study, candidemia was observed three times as much (31.6%). However, while antibiotics were started in all patients from the moment of admission, the clinicians did not have a common approach in terms of AF.

The IDSA guideline recommends empirical AF treatment for patients with a history of intra-abdominal surgery and anastomotic leakage [1]. Our study observed that the prophylactic or empirical AF treatment approach before the culture results was more dominant than the culture-based treatment approach (70% vs. 12.5%).

Holzknecht et al. reported that the administration of fluconazole prophylaxis after abdominal surgery reduced the rate of candidemia [17]. The IDSA guideline recommended echinocandins for empirical therapy in hemodynamically unstable, non-neutropenic, critically ill patients at risk for invasive candidiasis [1]. In the study by Bassetti et al., echinocandins were mostly used (64%) in the AF treatment [4]. In the non-invasive observational study by Lee et al., it was reported that echinocandins were more frequently prescribed for empirical therapy, while azoles were more frequently preferred for culture-directed therapy [18]. In our study, fluconazole was the most used agent in both prophylactic and empirical treatment. However, it was observed that patients (31.6%) who received prophylactic and empirical AF required AF change due to clinical or microbiological unresponsiveness, and micafungin was the most frequently passed drug. In our study, it was thought that echinocandins might be preferred in prophylactic/empiric treatment for patients with abdominal contamination with a prolonged waiting period since non-albicans species were observed to increase as the waiting time until the operation with abdominal contamination increased.

Candidemia rates in blood cultures taken before AF were higher in the empirical treatment group than in the prophylaxis group (43.5% vs. 11.3%). Because of this result, empirical treatment decisions were considered to be more accurate in diagnosing candidemia. Bassetti et al. evaluated prophylaxis given after the first 24 hours as inadequate AF treatment and reported that it increased 30-day mortality rates [4]. Garey et al. also indicated that delayed initiation of AF therapy is associated with mortality [19]. Vergedis et al. accepted AF treatment given in the first five days as early AF therapy and found it to be associated with survival [20]. In the study by Lee et al. with critically ill patients diagnosed with intra-abdominal sepsis, 33 patients were administered early empirical and 19 patients were given culture-directed AF therapy. It was revealed that the early empirical treatment arm had low 30-day mortality and a shorter median time for clinical improvement [18]. In our study, the average time elapsed from the operation with the abdominal contamination to the prophylactic AF was 1.5 ± 1.6 days. The mean time from the onset of perforation/leak to the onset of prophylactic AF was 4.9 ± 4 days. No significant correlation was determined between these periods with candidemia, seven-day mortality, 14-day mortality, and 30-day mortality rates. In the empiric AF treatment, the mean time from the operation for abdominal contamination to the administration of empirical AF was 7.6 ± 9.5 days, and the prolongation of this period was found to be associated with the development of candidemia. The mean time from the appearance of perforation/leak to the onset of empirical AF was 9.4 ± 9.5 days, and this time was longer in patients with candidemia.

Although the study by Lagunes et al. reported that inadequate source control and inadequate AF treatment did not affect mortality [21], in the study of Yan et al., it was demonstrated that the mortality in the group that received focal control and appropriate AF treatment together was lower than the group that received appropriate AF treatment alone [3]. In our study, the increase in the waiting time until the operation was associated with candidemia, especially a waiting time of 4.5 days or more. The present study also determined that prolonging the waiting period until the operation increased not only candidemia but also accompanying bacteremia. However, no significant correlation was observed between waiting time and overall mortality rates of seven, 14, and 30 days and at any time during hospitalization.

Limitations

The study’s limitations include the heterogeneity of AF and antibiotic applications due to the study’s retrospective design, being a single-center study, and lack of a common approach.

## Conclusions

In this study, although preventive AF treatment did not reduce mortality, it was found that it reduced the rate of candidemia and shortened the hospitalization period. It was concluded that preventive (prophylactic/empirical) AF treatment can be administered, especially among patients who present with abdominal contamination and previous operation history, delayed admission to the hospital, or delayed surgical operation.
